# Route optimization as an instrument to improve animal welfare and economics in pre-slaughter logistics

**DOI:** 10.1371/journal.pone.0193223

**Published:** 2018-03-07

**Authors:** Mikael Frisk, Annie Jonsson, Stefan Sellman, Patrik Flisberg, Mikael Rönnqvist, Uno Wennergren

**Affiliations:** 1 Department of Physics, Chemistry and Biology (IFM), Linköping University, Linköping, Sweden; 2 School of Bioscience, Skövde University, Skövde, Sweden; 3 The Forestry Research Institute of Sweden, Uppsala, Sweden; 4 Département de génie mécanique, Université Laval, Québec, Canada; Universita degli Studi della Tuscia, ITALY

## Abstract

Each year, more than three million animals are transported from farms to abattoirs in Sweden. Animal transport is related to economic and environmental costs and a negative impact on animal welfare. Time and the number of pick-up stops between farms and abattoirs are two key parameters for animal welfare. Both are highly dependent on efficient and qualitative transportation planning, which may be difficult if done manually. We have examined the benefits of using route optimization in cattle transportation planning. To simulate the effects of various planning time windows and transportation time regulations and number of pick-up stops along each route, we have used data that represent one year of cattle transport. Our optimization model is a development of a model used in forestry transport that solves a general pick-up and delivery vehicle routing problem. The objective is to minimize transportation costs. We have shown that the length of the planning time window has a significant impact on the animal transport time, the total driving time and the total distance driven; these parameters that will not only affect animal welfare but also affect the economy and environment in the pre-slaughter logistic chain. In addition, we have shown that changes in animal transportation regulations, such as minimizing the number of allowed pick-up stops on each route or minimizing animal transportation time, will have positive effects on animal welfare measured in transportation hours and number of pick-up stops. However, this leads to an increase in working time and driven distances, leading to higher transportation costs for the transport and negative environmental impact.

## Introduction

Meat production has increased rapidly around the world in the past decades. This increase has had a major impact on the environment, animal welfare and global trade [1–3]. In Sweden the meat industry produces 133 100 tons of beef, 233 500 tons of pig meat and 5 100 tons of sheep and lamb meat per year [4], representing more than three million animal transports from farms to abattoirs per year. Transportation and handling are essential factors in the pre-slaughter production chain, playing an important role in animal welfare, meat quality and the risk of disease transmission [5]. The pre-slaughter logistics chain comprises the transport operations and includes scheduling, management and control of animals transported from farms to slaughterhouse [5–7]. As described in Ljungberg et al. [5], animals are often collected from many farms. The logistic process include potentially stressful factors such as road conditions, climate, traffic conditions, number of pick-up farms, transportation time, and distance and queuing at abattoir. The factors expose the animals to various stimuli such as increased human contact, transportation, unfamiliar environments, food and water deprivation, social structure changes and climate changes that may lead to fear, dehydration, hunger, increased physical activity, fatigue and injury [8].

Efficiency in animal transportation is not only important from an economic and environmental perspective, but also important for animal welfare [7, 9–14].

One way to reduce negative effects of livestock transportation is to minimize the average transport distance. Short transport distances have obvious advantages such as lower costs and fewer emission. Another advantage includes a route to improve animal welfare in pre-slaughter logistics. Additionally, shorter animal transport distances reduce the number of animal injuries [15–17], while longer distances increase susceptibility to diseases and poor welfare outcomes [9, 11, 18–20]. Mortality increases when distances exceed 100 km [10, 12]. What’s more, several studies have shown that transportation time affects meat quality [21–23]. Villarroel et al. [24] reported that transport time affected meat quality in terms of tenderness and overall liking. Ferguson, Warner [8] reported that pre-slaughter stress can have a detrimental effect on meat quality in beef and lamb.

Longer transport times however, must not always be linked to reduced animal welfare. Some studies have shown that short journeys can be more stressful than long journeys for pigs and horses [22, 25]. Journey conditions, such as road quality, density, driving style, vehicle design and routing are important factors for animal stress levels [26–30]. Although Nielsen et al [27] and Cockram [28] point out that long duration journeys compromises animal welfare, duration does not contribute to negative welfare; instead, time-based factors such as temperature, lack of food, water and rest contribute to this welfare. Still, this raises the need for discussion about journey duration: the shorter the journey, the lesser the effect on animals.

Aside from transportation time, the number of pick-up stops for loading along each route from farm to abattoir is crucial for animal welfare, since animals are confronted with unknown individuals during the loading process from other farms [7, 31, 32]. Confronting new animals can lead to increased stress level and agonistic behavior [31, 33], and loading animals at many farms along the same collection route increases the risk of disease transmission; hence, the quantity of farms visited and their sequence are of paramount importance [28, 32, 34–37]. When the number of pick-up stops are limited, the risk of disease transmission is lower. Group loadings are limited by regulations outlining how various animal categories and types can be mixed aboard a vehicle or within the same vehicle compartment, how vehicles should be configured, or how farm visitations should be carried out due to health status.

Typical registration and planning processes, vehicle fleets and other important parts in the pre-slaughter logistic chain are described in [5, 7, 34, 35].

The potential route options, including pick-up dates and number of pick-up stops, are extensive, and the use of logistic tools for optimizing animal transports have a large potential for improving animal welfare and reducing costs [34, 35, 38, 39]. Many existing optimization models and earlier analyses, however, are too simplified and demand further development. For example, the model presented by Gribkovskaia et al. [34] cannot be used in case studies with big datasets due to long solution times. Oppen et al. [35] deals with only two weeks of real life data and one slaughterhouse. Håkansson et al. [39] use a large set of real data, but are only used for strategic analysis with an optimization model based on a facility location model and no detailed route calculations. We have not found any study in animal transportation where optimization is used to analyze large datasets for operative planning or regulations that can affect stressors like animal transport time. In addition, no study evaluates how different transportation planning conditions, such as the planning time window length or transportation time restrictions and the number of pick-up stops, affect overall transportation time.

In this study, we have used an optimization model and one-year animal slaughter transportation data to examine two key stress factors: animal journey time and journey length in different scenarios. The scenarios focus especially on *i*) the length of the planning time window, *ii*) reduced journey distances and time, *iii*) the reduction of pick-up stops and *iv*) the limited transportation time along each route. We have further developed a model used for route planning in forestry [40] designed it to handle the characteristics of animal transports. Some differences are the increased number of pick-up stop in each route (in forestry this is typically one), different loading and unloading characteristics, and new route time limitations. An important scientific contribution of this paper is the detailed analyses on live animal transportation route problems using a large set of registered transports in Sweden. Another contribution is the modified route solution method that solves planning problems efficiently. This is based on a tabu search method, where special consideration must be taken regarding the multiple animal pick-ups within each delivery and new limits on route times.

## Materials and methods

In the analysis we compare key figures in animal transportation related to animal transport time, distance and number of pick up stops in each route for six scenarios. The base scenario is the first of six that uses real 2008 data on cattle transports to the five largest abattoirs in Sweden. The data includes daily information about the amount of cattle that have been transported from a specific farm to a specific abattoir. We denote each date, farm and abattoir combination as a transport occasion. For example, if the data reports that fifteen cattle were transported from farm X to abattoir Y on January 1, 2008, we have a transport occasion. Five simulated scenarios are defined from the base scenario. These scenarios are formed using different combinations of two transport constraints: maximum number of pick-up stops along each route and maximum animal transportation time. Both are directly associated to animal welfare. All scenarios must include all transport occasions. That is, the fifteen cattle in the example must be transported from farm X to abattoir Y in one transport occasion in each scenario.

Additionally, we compare results from optimizations carried out with one-day, seven-day and fourteen-day planning time windows. These windows are used to represent the possibility of changing the choice of pick-up day for each transport occasion. For example, when using the one-day time horizon, all transport occasions must be performed by the optimization model on the same day. When using a seven-day time horizon, however, all transport occasions carried out must be performed by the optimization model within the same week, though the actual day within the week is changed.

### The route optimization model and solution method

**Fig 1:**
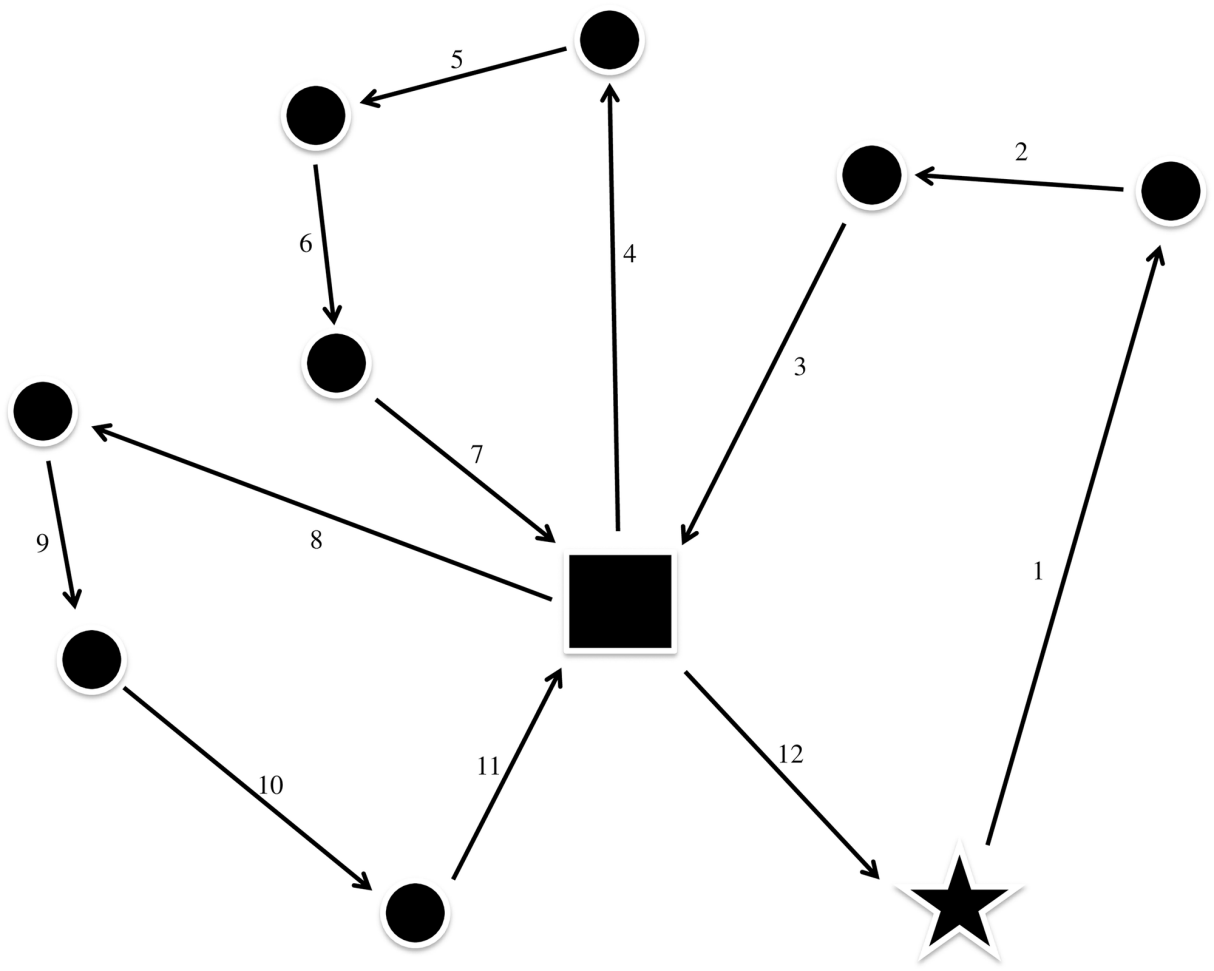
Description of the three routes taken by one truck for one day. The numbers on the arcs indicate the order of driving throughout the day. Circles represent farms, the square represents an abattoir, and the star represents the driver’s home base.

Our model and method are built on the RuttOpt route optimization system developed for forestry with timber truck transport as described in [40] and [41]. Pick-up and delivery vehicle routing are the general problems for this system. Fig 1 illustrates the routing problem for one day and one truck. The truck starts in its home base and drives to the first farm where it is loaded. Next, it drives to the second farm, where it completes the first trip, drives to the abattoir, and unloads the animals. Before the truck returns to the home base, it makes two additional trips, each to three farms. There are several restrictions to consider for each truck. Below are a few.

- Each truck has a limited animal capacity; this varies depending on animal commercial class.
- Each truck has a daily working schedule.
- Each farm must be visited within a certain time window.
- All transports to the abattoir must be within a certain time window.
- Driving time along each route is limited from the first farm pick-up to the abattoir.
- The number of animal commercial classes that can be transported together is limited.

The objective for the route optimization model is to minimize transport cost, defined as the sum of all monetary costs for using the truck in animal transport, including driver wages, fuel, wear, depreciation, service and maintenance. The cost used in the optimization model is based on contracts between haulers and livestock truck companies, representing the actual transport cost for the abattoir company.

The solution method in the forestry model is based on two phases. In the first phase, a Linear Programming (LP) model is used to make an optimal allocation of log flows from supply point (harvest areas) to demand points (mills). In the second phase, a modified tabu search method is used to solve a vehicle routing problem based on so-called transport nodes. In the forestry application, a truck is typically loaded at one supply point and takes a full truckload directly. In this case, a transport node is described by the full delivery from a supply point to a demand point. Our animal transport model has some main differences. First, the animals’ destination to the abattoir is already decided, making the first phase useless. Second, the generation of transport nodes is different, for we typically have multiple pick-ups.

**Fig 2:**
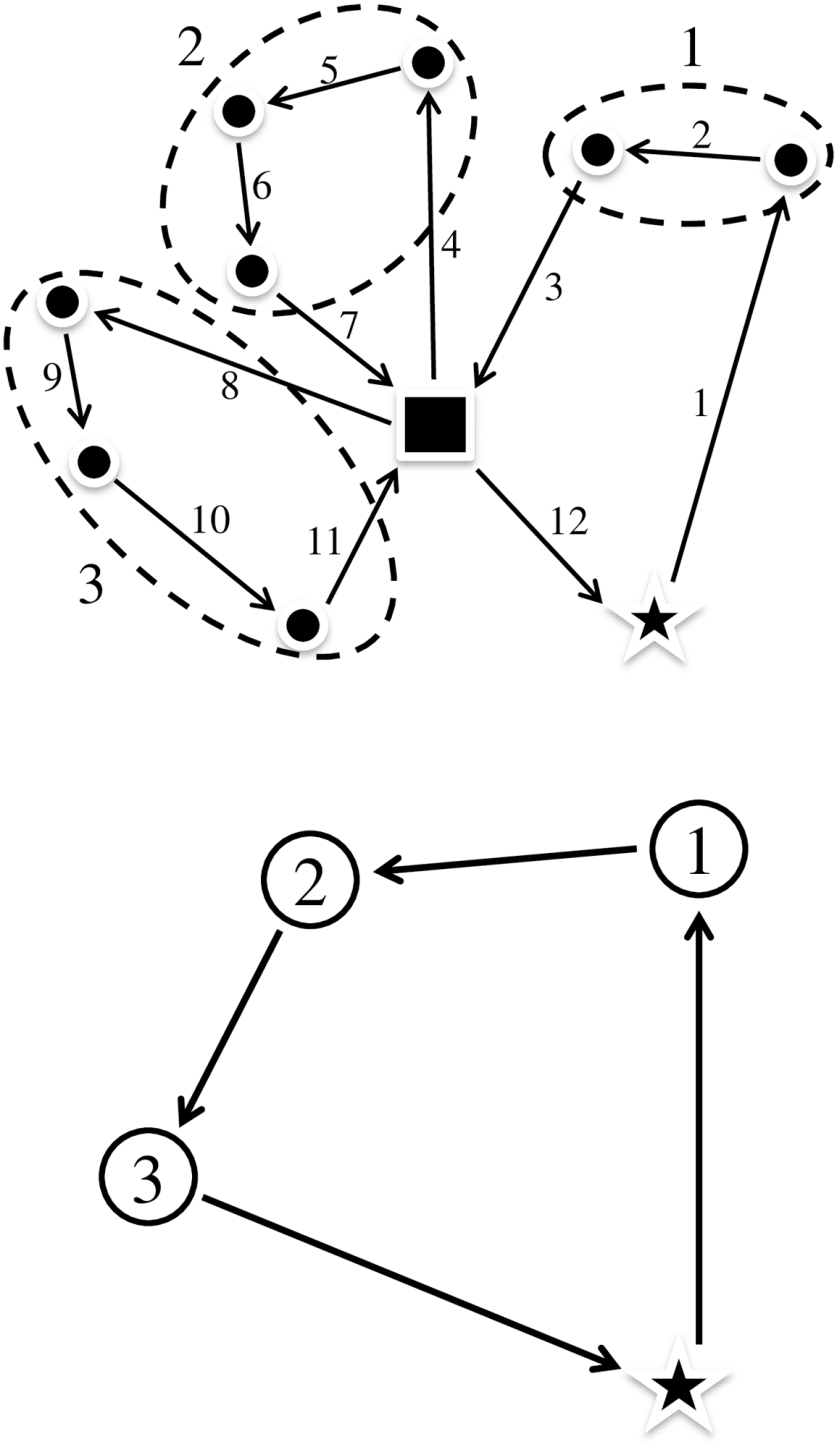
Illustration of how three farm routes are combined (left) to form three transport nodes (right).

Fig 2 provides an illustration of the transport node process forming in our model. All farms visited along one route before abattoir delivery are merged to form a transport node. A transport node is generic and includes information on farms that are included. Each transport node represents all pick-ups and deliveries; therefore, we only need to sequence the transport nodes. It is possible to combine nodes for many trucks and solve a standard vehicle routing problem [42]. This can be done in many ways. First, nearby farms can be combined into an aggregated farm group. It would be vital to ensure that truck capacity is not exceeded. Starting the generation with the farthest farms and adding nearby ones along the route would be a straightforward approach if the total distance to the abattoir does not exceed a set threshold value. Second, we can solve a generalized assignment problem with an integer program. Both approaches perform similarly in our analysis. We have combined these with limits on the number of stops at farms in the results. These limits are easy to implement as it is done a-priori when the transport nodes are generated.

To solve the original forestry problem we use an extended version of a general tabu search method, where we added some functions not found in the original version developed by [43]. The added functions handle differences in animal supply at farms and animal demand at abattoirs and more home bases. The latter represent the starting point for trucks. More details are found in [41]. We have additional features for animal transports regarding multiple pick-up stops and route time limits described earlier.

The model requires information on distances among all farms and between farms and abattoirs. In general, the best possible route between two farms or to the abattoir may not be the shortest route. Instead, a combination of distance, road quality, curves, hilliness, and speed limits provides the best possible route. To compute these combinations, we have used the Calibrated Route Finder route calculation system [44]. This is used by the Swedish forest industry to establish fair and balanced distance calculations for heavy trucks. In addition, it is used for invoicing among different parties. The data is taken from the Swedish National Road Database forest version; the data includes detailed information on all the country’s public and private roads. One must only have contact information from farms and abattoirs to calculate correct distances.

### Scenarios and simulations

The six different scenarios used in our analysis are described as follows:

1. Base: a real-life scenario with constraints as current regulations for animal transport in Sweden.
2. Half time: the allowed time for an animal to be in transport was reduced to half.
3. Max 3 stops: the maximum number of pick-up stops allowed along one route was set to three.
4. Max 2 stops: the maximum number of pick-up stops allowed along one route was set to two.
5. Max 1 stop: the maximum number of pick-up stops allowed along one route was set to one.
6. Welfare: the allowed transport time was reduced to half, giving way to two allowed pick-up stops.

For each of the six scenarios we made three optimizations representing three planning time windows: one day, seven days and fourteen days. Through the optimization results, we have summarized key factors such as animal transportation hours, total driving time, total distance, total working time and the number of animals subject to varying numbers of pick-up stops. On one hand, animal transportation hours and animals subjected to many numbers of pick-up stops will influence animal welfare. On the other hand, driving time, distance and working time will influence economics and environment. All optimizations comprised data of one-year cattle transportation to five abattoirs.

### Data

**Fig 3:**
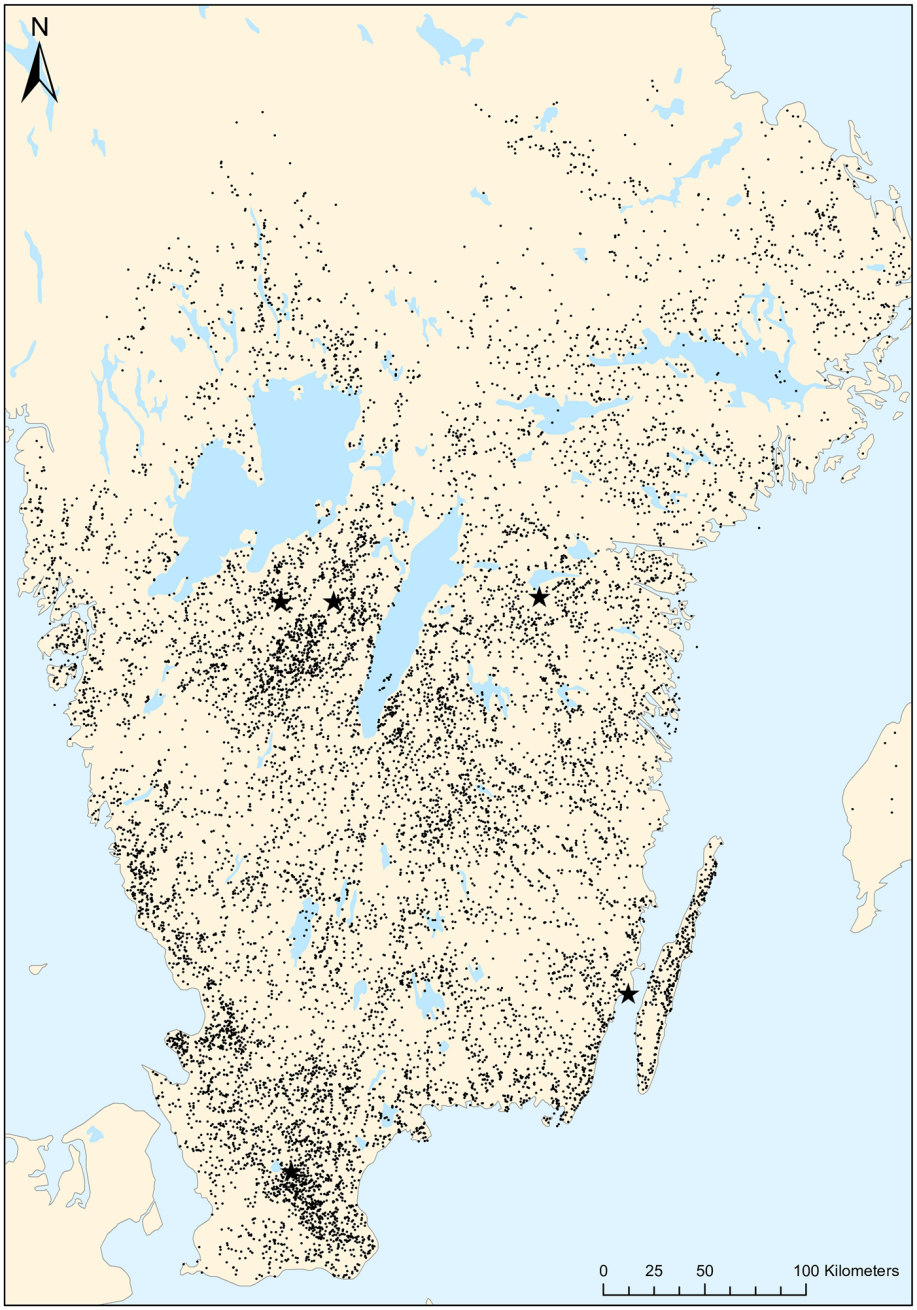
Geographical extent of the analyses. Black stars represent the abattoirs and grey dots represent the farms. Made with Natural Earth.

The data was provided by the Swedish Board of Agriculture and included information on transportation between farms and abattoirs as well as dates and quantities of animals transported. The study was limited to cattle transport to the five largest abattoirs in 2008, which was capped at 58%. Analyses were explicit for each abattoir, though our results are the sum for all abattoirs combined. Of the 12 396 farms, we could not find addresses or coordinates for 566 areas of land. Instead of excluding these farms, we gave them the same coordinates as a farm randomly chosen in the same county. The geographical extent of the analyses with 12 396 farms and five abattoirs is shown in Fig 3.

**Fig 4:**
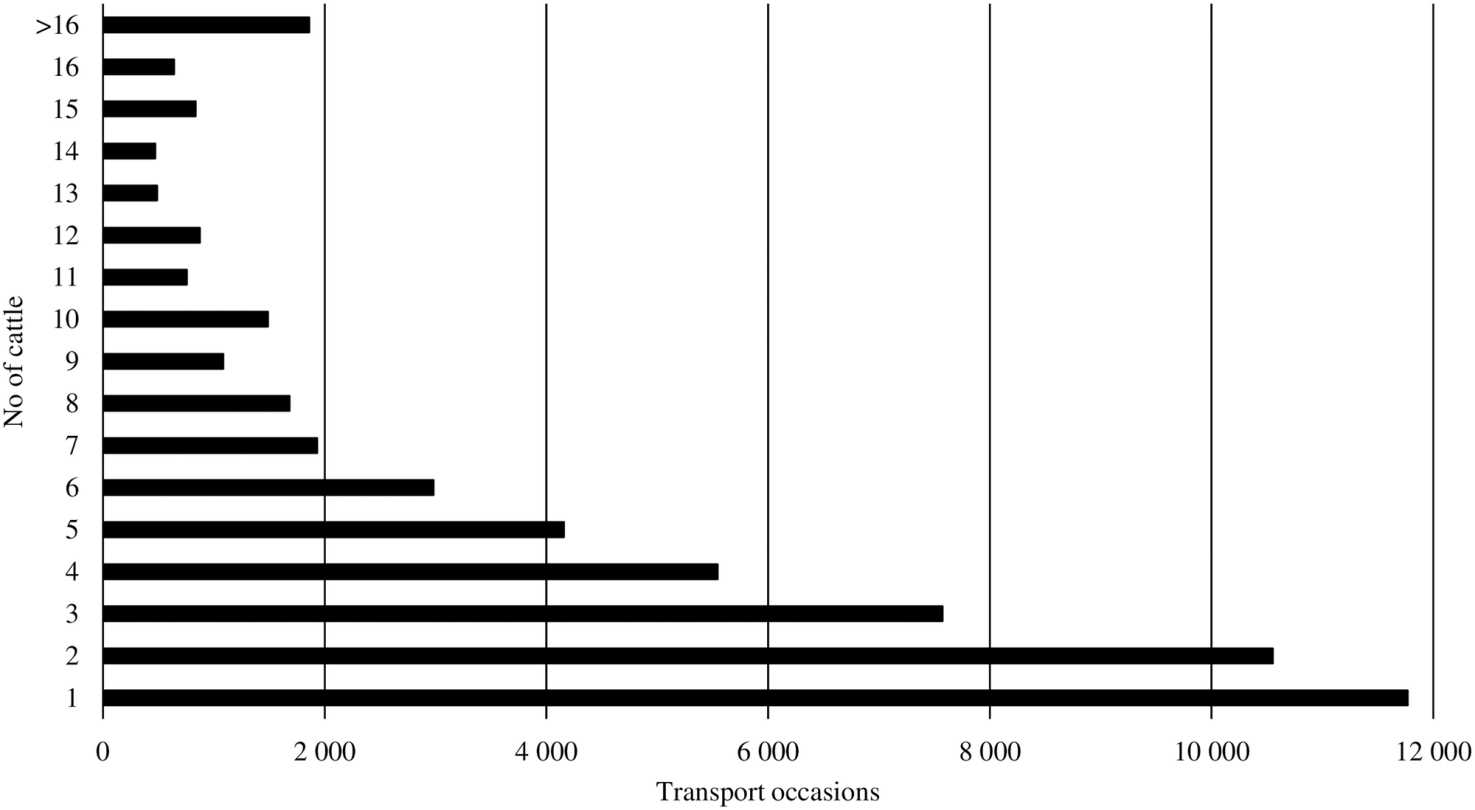
Number of transport occasions classified by the number of individual cattle transported at each occasion.

The total number of cattle transported was 265 956. Each farm had an average of 4.9 cattle available for transport on each occasion. The total number of transport occasions was 54 702. Fig 4 presents how many cattle were reported at each transport occasion.

The data did not include any routing or truck capacity information. Only information about farm id, abattoir id, number of animals and date for transport occasions was available. Thus for the base scenario, we had to construct routes that most likely simulated the real routes. This was done by having the route optimization model optimize all transport occasions for each separate day. According to the transportation data, if two nearby farms had delivered cattle to the same abattoir the same day, it would have been assumed that these two transport occasions were part of the same route. When the model optimized each separate day, it automatically combined the two transport occasions if the combination represented a lower transportation cost than the corresponding single transport occasion. The model would have most likely created realistic simulations of the actual routes for each day.

Moreover, given the information available, it is possible that the created routes are better than reality, since the optimization model would find the optimal solutions. The real routes were manually planned and were unlikely to be the optimal solutions. Thus, the solution created by the model is an upper limit to how well the transports were performed.

**Fig 5:**
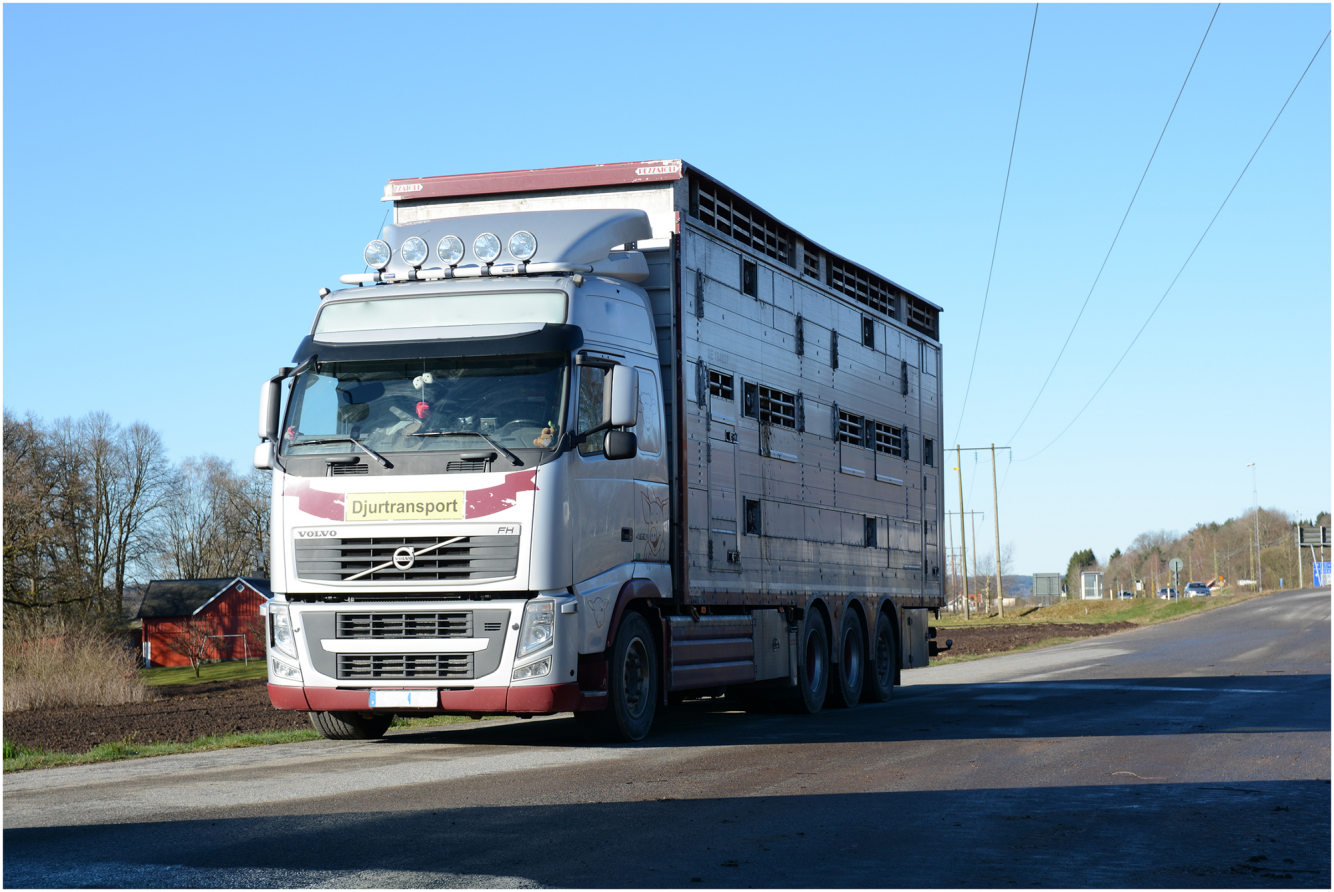
A typical Swedish livestock truck used for cattle transport.

A typical Swedish livestock truck used for cattle transport is illustrated in Fig 5. Truck capacity is normally fifteen or sixteen cattle, but some variation between trucks exists. Trailer trucks can normally carry between thirty and thirty-two cattle, though it is possible for these vehicles to allow a maximum capacity of fifty-five cattle. Trucks are usually loaded from behind. The loading area can be divided in several pens to transport animals that cannot be mixed according to regulations. Loading density influences both welfare and economics [7, 23]. Further, Swedish regulations define the maximum loading density as minimum area per animal. For cattle (325 kg), the minimum area needed is 0.4–2.7 m^2^ per animal depending on weight. For average cattle, the area is 1.3 m^2^ per animal. [45]. Journey lengths should influence animal transport density [31]. Weather conditions, size, species, weight, journey duration, truck standard, road conditions, however, make stating rigid recommendations on stocking density difficult.

**Fig 6:**
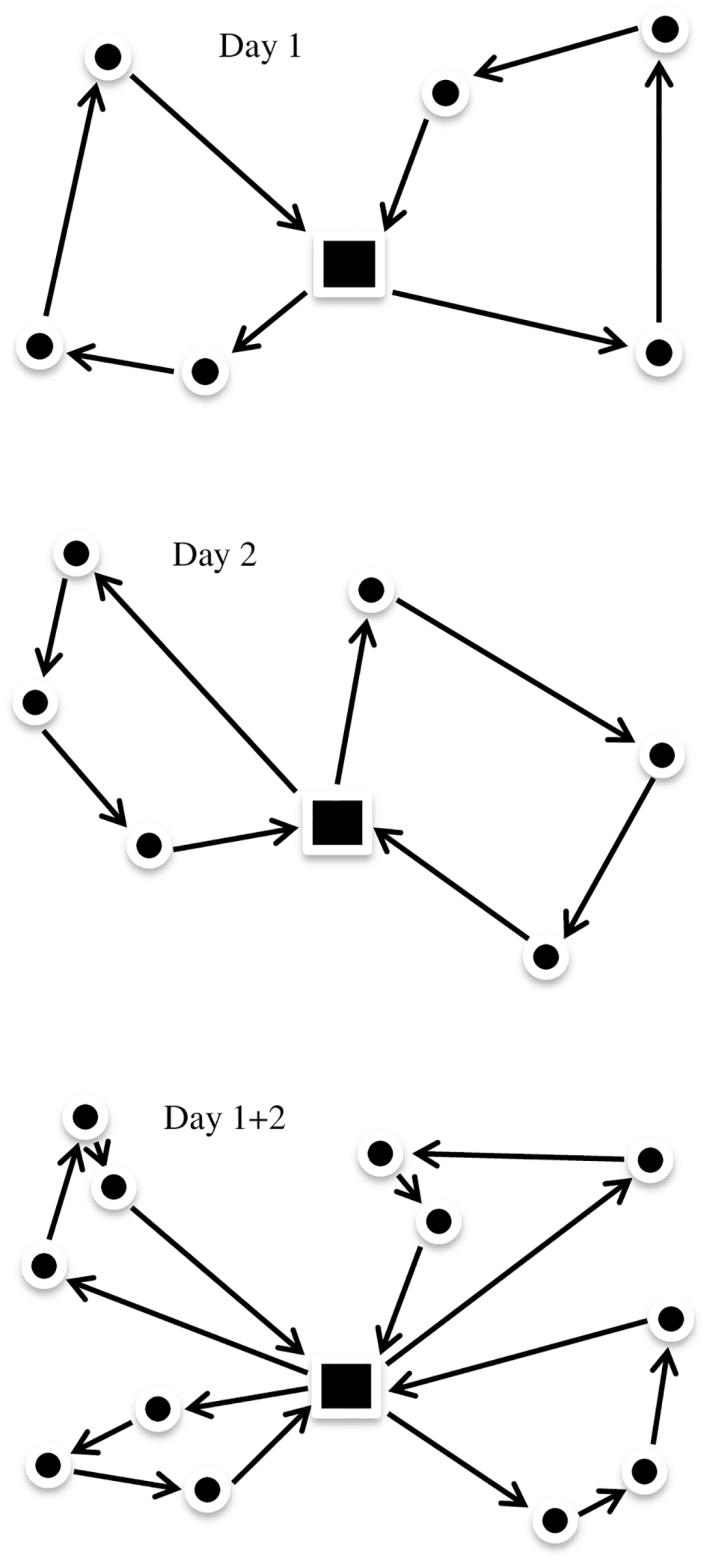
Example of daily optimized routes (left and center) and an integrated two-day optimization (right).

One-day, seven-day and fourteen-day time horizons were tested in our simulations. The alternatives with seven and fourteen days represent the possibility to find better routes compared to routes performed in real time. This is done by combining pick-up places that were visited during a seven- or fourteen-day period. Fig 6 illustrates the potential for more effective optimization with a broaden time window for deliveries. In this example, we assume that the capacity is such that three farms can be combined to one full truck delivery. Clearly, better routes are found when there are more farms to choose from.

In the Half time and Welfare scenarios (with reduced allowed transportation time), we excepted transport time constraints for farms distances to abattoirs that would make transport impossible. Not doing so would have resulted in varying numbers of transported animals between scenarios, making comparisons difficult.

### Parameters

Most parameter values were retrieved from a case study on slaughter transport logistics at two Swedish abattoirs performed by Algers et al. [46]. The trucks were assumed to be available between 6:00 am and 12:00 pm but with an extra cost if used after 3:00 pm. Farms and abattoirs were open for pick-up and delivery night and day, making truck availability the limiting factor for transport. The individual truck capacity was defined to fifteen cattle and was assumed to be the same for all trucks. To prevent the number of trucks from becoming limiting factor, we decided to make thirty trucks available, resulting in an overcapacity. Additionally, prior to loading, we set the loading time to ten minutes to arrange trucks and make other preparations. Lastly, we factored in three minutes to load each cattle. A full truck load of fifteen cattle would require a load time of fifty-five minutes. Twenty minutes were allotted to unloading. Loading time varies on many factors, including animal state (are they kept loose or tied at the farm?), human manipulation and farm conditions. These factors are combined with estimated unloading time through discussions with slaughter company personnel. The same loading times are approximately measured in field trials and described by Aradom et al. [19]. It is generally easier to unload at slaughterhouses than loading at farms because of practicality, resulting in shorter loading times.

The truck transport cost was set to 89.5 Euro per hour for loaded driving and 72.1 Euro per hour for empty driving. The cost difference is due to fuel consumption. Overtime transport after 3:00 pm is ten times the original cost. This does not represent real overtime cost, but it gives the optimization model the possibility to pick up cattle from farms located remotely from abattoirs and limit the use of overtime in the model’s route creation.

The minimum number of cattle to be picked up in a single route was set to one, while the maximum number of farms to visit in one single route was set to eleven (i.e., eleven pick-up stops). The number of stops is not regulated; one and eleven are considered extreme numbers. In fact, one of the routes in Algers et al. [46] visited eleven farms, making this figure a maximum number. With the exception of the Half time scenario, whose maximum time for animal transportation was four hours, the maximum time was eight hours in the other scenarios. The time limit includes loading the first animal and unloading the last animal. If a farm was remotely located from an abattoir that exceeded the maximal allowed time, however, the constraint was released as per applicable regulations in Sweden.

Data from real transport occasions has been used in the simulations. Hence, one-day optimizations would have been entirely possible in real time. But some of the optimized routes would not have been possible had the seven- and fourteen-day time spans been applicable, since cattle types are slaughtered during specific days of the week. Because we did not have data for loaded animals in specific trucks, we could not take slaughtering schedules into account making it impossible to compare real-life situations with simulations. We did, however, make comparisons with the various optimized scenarios. Moreover, trucks were assumed to use the same principle as the Calibrated Route Finder [44] to find the best route between nodes in the road network.

All runs have been done on a standard PC with an i5 processor containing 3.3 GHz and 16 GB of internal memory.

## Results

### Effect of planning time window

The planning time window length has a substantial effect on the calculated key factors: driving time, distance and animal transportation hours. Table 1 shows the sum of the transport key factors, namely animal transportation hours, total driving time, total distance and total working time for the Base scenario. The working time has varying lengths of planning horizons set at one day, seven days, and fourteen days. To calculate the number of animal transportation hours, we multiply the transport time (h) by the number of animals. For instance, if fifteen cattle are transported from point A to point B in two hours, we should have a total of thirty animal transportation hours. Broadened planning time windows decrease the working time and the other key factors, creating the highest impact on distances and driving time. The difference between a one-day and seven-day time horizon implies a potential reduction of animal transportation hours at 2.6%; the distance and driving time are capped at 9.9%, and working time is set at 8.3%. If the time horizon is spread out over fourteen days, the difference from a one-day time horizon is 4.3% for animal transportation hours, 12% in total distance and driving time, and 11% in total working time.

**Table 1:** Animal transportation time, total driving time, total distance and total working time for the Base scenario, comparing the different planning time windows.

	1 day	7 days	14 days
**Animal transportation hours (h)**	713 055	694 478 (-2.6%)	682 038 (-4.3%)
**Total driving time (h)**	101 723	91 630 (-9.9%)	88 908 (-12.6%)
**Total distance (km)**	5 963 588	5 371 471 (-9.9%)	5 223 878 (-12.4%)
**Total working time (h)**	131 887	120 899 (-8.3%)	117 733 (-10.7%)

The results imply that route optimization has a potential for reducing animal transport hours, distance and working time.

### Effects of limiting animal transport time and number of pick-up stops

In the simulated scenarios, we imposed restrictions on allowed animal transport time and number of pick-up stops on each route. Optimization results show that these possible regulators may have a significant impact on transport factors essential for animal welfare, economics and environment.

#### Transportation time

**Fig 7:**
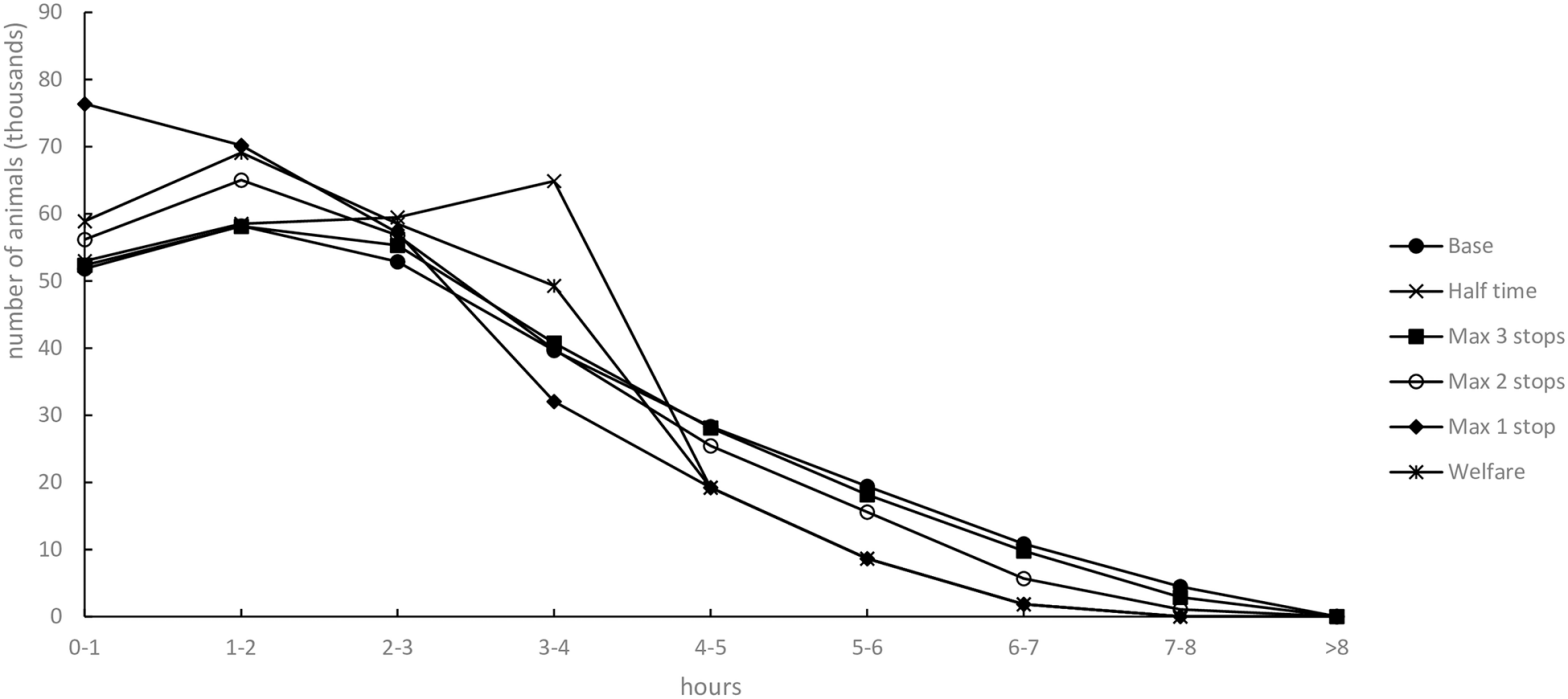
Animal transportation time for scenarios with a seven-day planning time window.

When considering individual animal time in transportation tested for the seven-day planning time window (Fig 7), the Base scenario gave the highest share of long transports and the least share of short transportation times. The Welfare scenario had the lowest share of long transportation times. Like the Welfare scenario, the Half time scenario had a low share of long transport times, but had more animals within the three- to four-hour interval and fewer animals in the lowest time intervals. The Half time scenario shares the same curve as the Max 1 stop and Welfare scenarios, since they are both characterized by transports exceeding four hours. These scenarios share the same curve because numerous transport occasions must be carried out with special dispensation if farms are more than four hours away from the abattoir. The dispensation has the same relevance for the Welfare scenario. Owing to time limits, the dispensation has similar effect on long-distance farms in the Max 1 stop scenario (no more than one farm can be visited if transportation distance exceed four hours). When compared to the Half time scenario, the Base scenario results in 14% more animal transportation hours.

#### Number of pick-up stops

**Fig 8:**
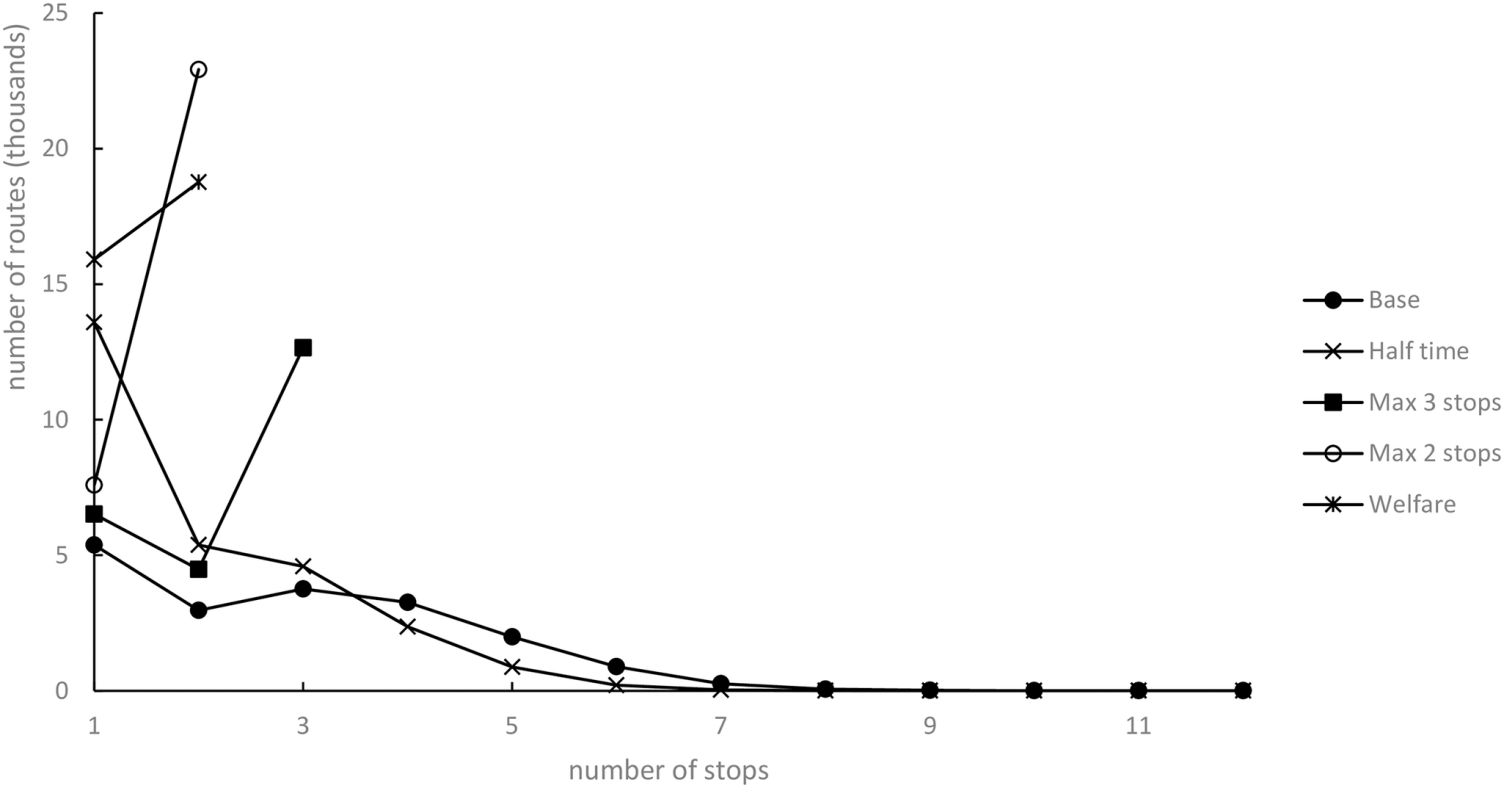
Number of stops along each route for scenarios with seven-day planning time window.

The number of routes with many pick-up stops is higher for the Base scenario than the other scenarios tested in the seven-day planning time window (Fig 8). This observation signals that the Base scenario is the worst from an animal welfare perspective. The quantity of routes with pick-up stops is less in the Half time scenario due to the model’s restriction on finding shorter routes. The other scenarios are determined by the maximum number of stops, thereby not resulting in routes with many stops. Few routes include more than six pick-up points in Base scenario. Less than 2% of the routes (346 of 18 561) in the seven-day planning time window have more than six pick-up points. Only 6.7% of the routes (1 238 of 18 561) have more than five pick-up points, while the Welfare scenario resulted in many more routes (+110%) with only one stop when compared with the Max 2 stops scenario. Both scenarios have set a maximum number of stops to two. In the Welfare scenario, however, the reduced animal transportation time will force the model to find shorter routes with fewer stops.

Our scenarios comprising limited pick-up stops can increase animal welfare by less confrontation with new animals and less risk of disease transmission, though the restriction does not have the same effect on the number of animal transportation hours as scenarios characterized by limited transportation time.

The Max 2 stops scenario provides a 11.2% animal transportation hour reduction, an increase to 4.6% of working time and 37.4% of driving distance. This scenario implies a reduction of disease transmission, for only two farms are visited along each route.

#### Distance and working time

**Fig 9:**
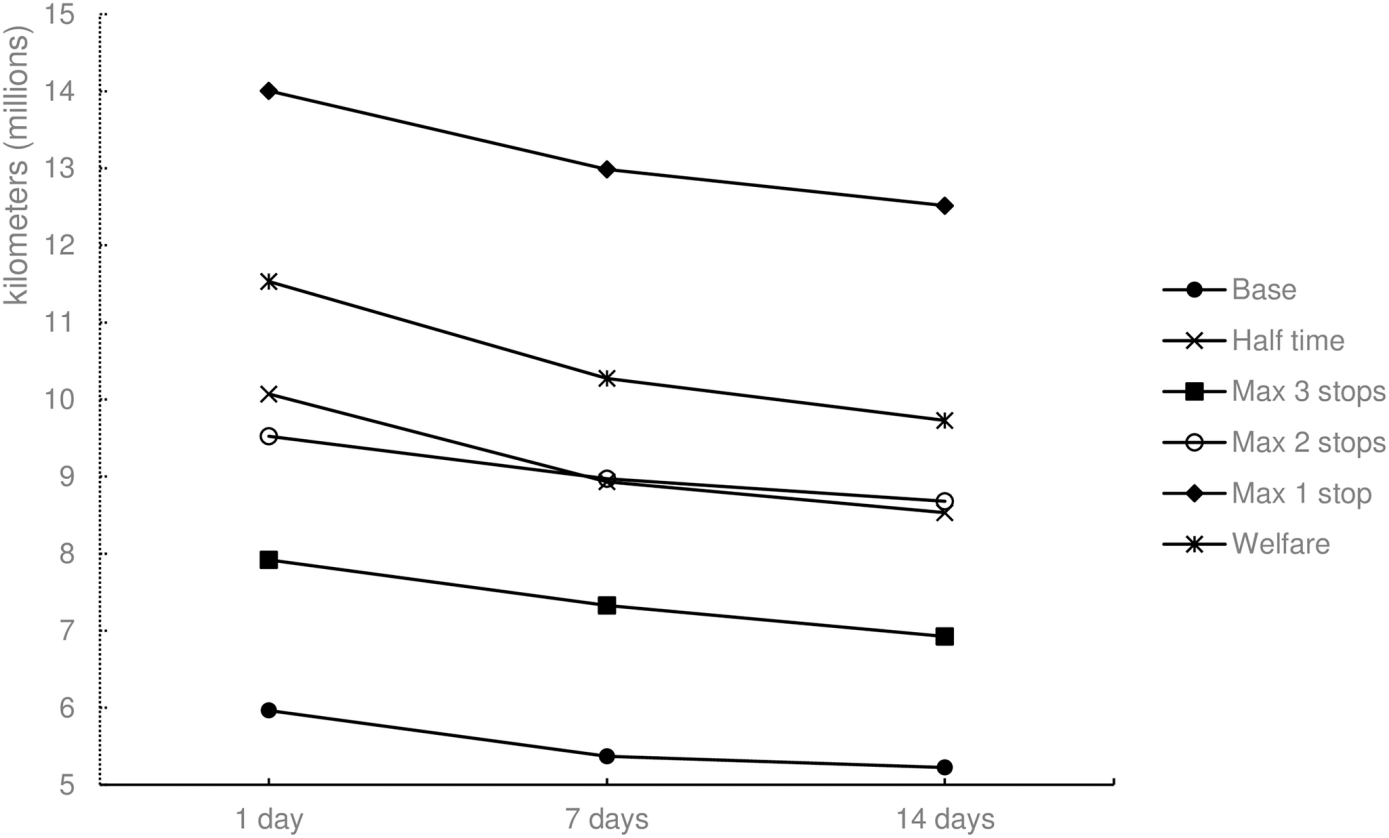
Total driving distance for the different scenarios and three planning time windows.

**Fig 10:**
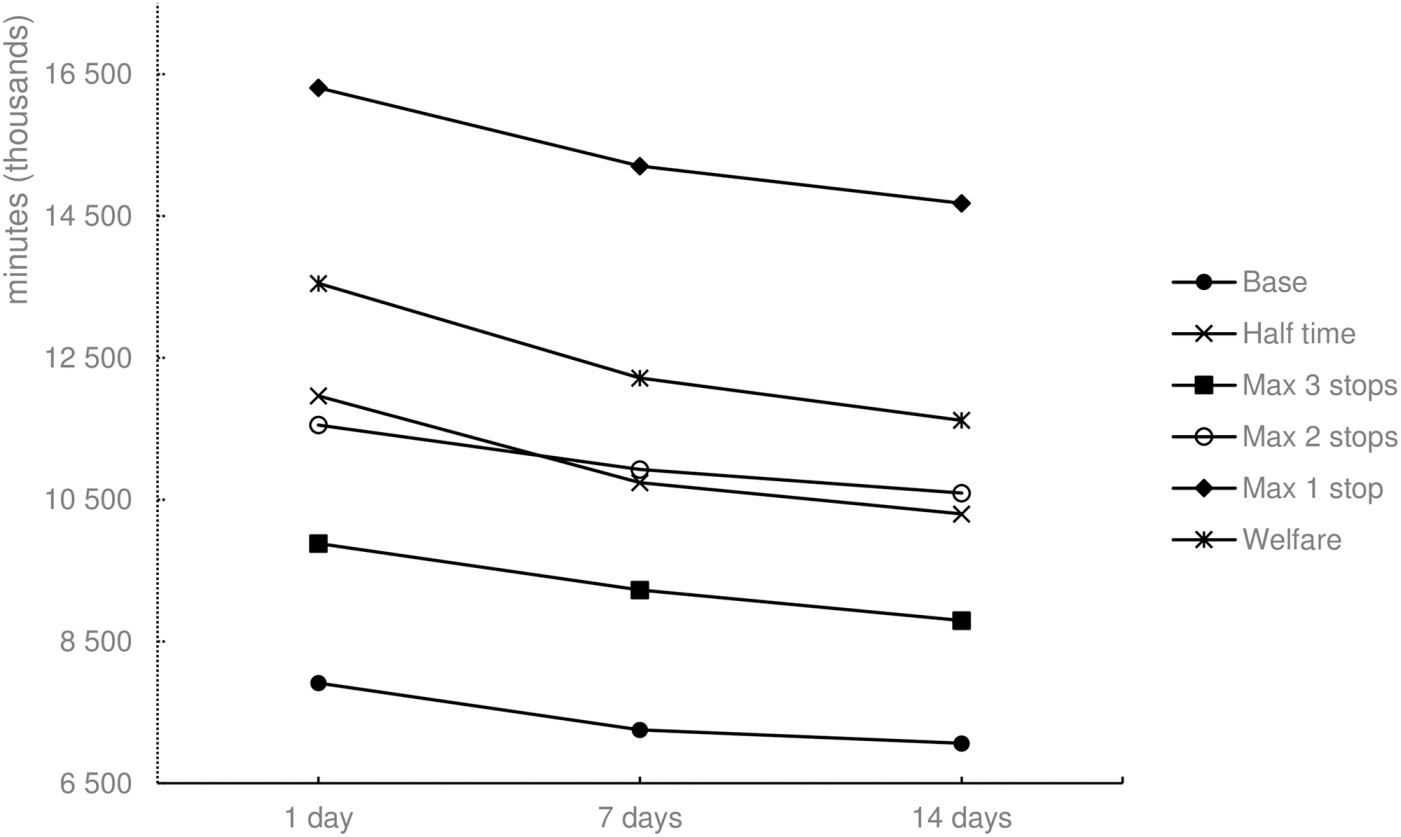
Total working time for the different scenarios and three planning time windows.

Unlike the Max 1 stop and Welfare scenarios (Figs 9 and 10), the Base scenario provides less driving distance and hauler working for trucks are used more efficiently and results in less time for delivering the same amount of cattle. With respect to the seven-day planning time window, the difference between the Base and Max 1 stop scenarios is more than 100%. The Max 2 stop and Half time scenarios yield approximately the same result with respect to working time and driving distance. When compared to the Base scenario, distance and time are approximately 67% higher in the two aforementioned scenarios, achieving approximately the same result as the transportation restraints along each route

#### Animal transport hours

**Fig 11:**
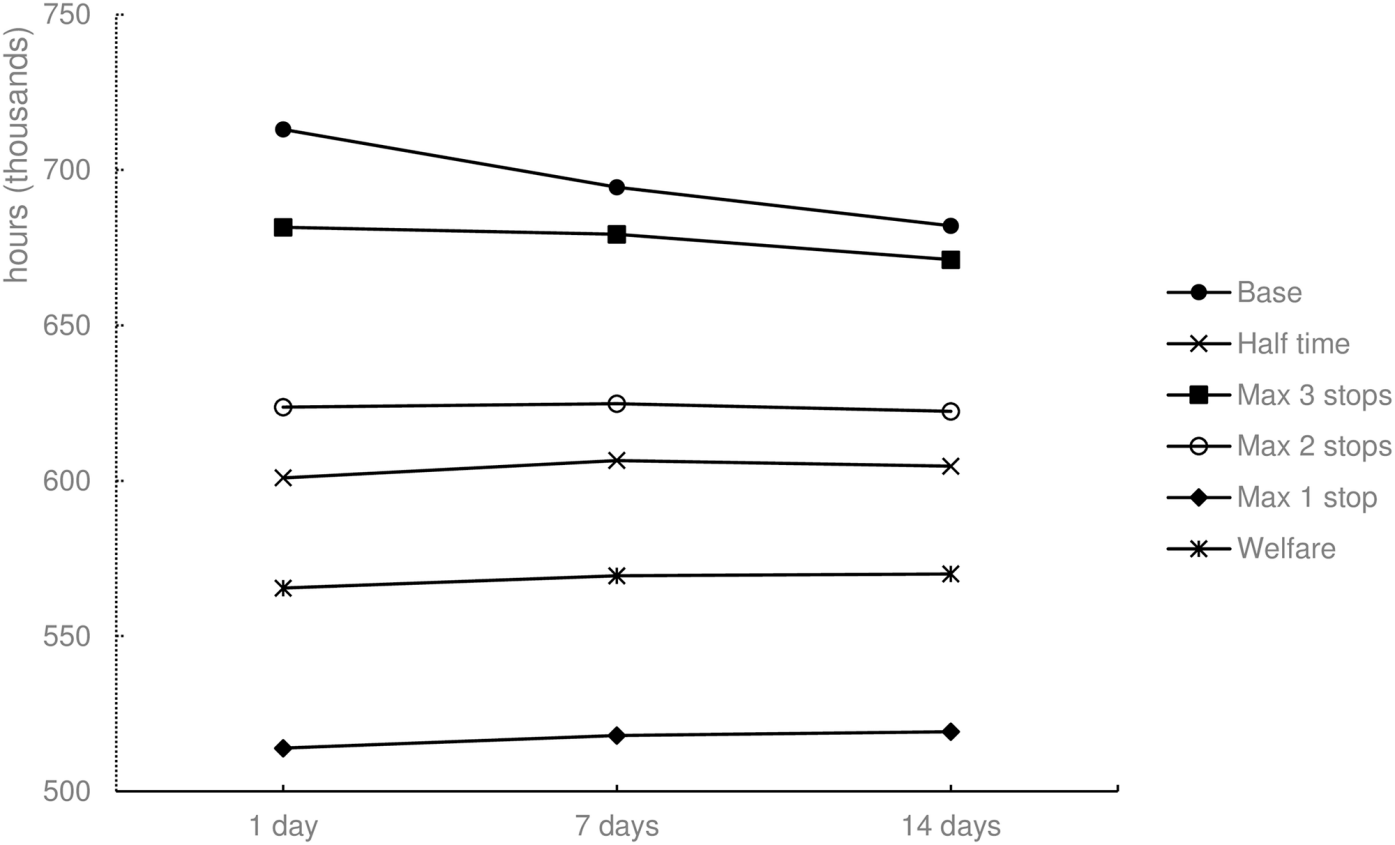
Animal transportation hours for the different scenarios and three specific time horizons.

Except for the Base scenario (Fig 11), animal transportation hours are not affected by different planning time window lengths since the available number of accessible cattle limits the transportation efficiency. The longer the planning time window, the more cattle can be chosen, resulting in more efficient route planning. The Max 1 stop scenario has the lowest sum of animal transportation hours because each route has only one pick-up. The Welfare scenario has the second lowest sum of animal transportation hours, since the allowed transportation time was limited to four hours; the number of allowed stops was limited to two. Finally, the Base scenario resulted in the highest sum of animal transportation hours, totaling almost 20% more hours than the Welfare scenario.

The results imply that a large effect in welfare, measured by animal transportation hours, can be achieved by limiting the journey duration for animals. If the allowed journey time is limited to four hours instead of eight, the reduction in animal transportation hours is 15.7% (Fig 11). The changes implies, however, that the total working time is increased by 51.5% (Fig 10), and the total driving distance is increased by 68.9% (Fig 9). This results in a high cost of animal welfare when measured by animal transportation hours.

## Discussion

We have further developed a pre-slaughter transport route optimization model combined with a large dataset of information covering one year of cattle transportation to five Swedish slaughterhouses. Transportation distance, working time, transportation work and number of farm stops along each route have significant impact on animal welfare.

We have shown that the optimization model measure and compare the effects various scenarios have on key welfare parameters. These can be related to the costs needed to achieve them. Our analyses show possibilities with condition changes, e.g., pick-up date (the time horizon), transportation time and the number of loadings along one route (number of stops). The simulation results show that the planning time horizon length has a significant impact on transportation time, driving time and total distance, all corresponding to economic and environmental effects on slaughter companies, haulers, and personal health and well-being.

Additionally, the results illustrate that animal transportation time regulation and quantity of pick-up stops can be incorporated in computerized route optimization. Because route optimization for animal transportation can reduce distance and driving time, transportation costs and emissions may likely be reduced as well. Economic and environmental effects need to be evaluated; it is imperative to strike a reasonable balance between animal welfare, the haulers’ wellbeing, and the economy. Currently, we cannot say which regulations will lead to the highest increase in animal welfare; this will require further research.

Our study is limited to cattle transportation in Sweden. However, the results should be transferable to other species and areas for which the planning process and transport conditions are similar.

Our results show that increased animal welfare in the pre-slaughter logistic chain may lead to increased costs. Dawkins [47] concludes that a high standard of animal welfare can enhance commercial value because of reduced mortality, improved health, product quality and disease resistance, reducing potential conflicts between animal welfare and production. However, future research and new animal welfare optimization strategies are needed.

Carlsson et al. [48] investigated consumers’ willingness to pay for welfare friendly products. This did not require animal transportation, since slaughters were carried out at farms via mobile abattoirs. One conclusion confirmed that Swedish meat consumers had a positive willingness to pay for cattle slaughtered in mobile abattoirs instead of larger slaughterhouses. In that survey, 39.5% of respondents were willing to pay for mobile abattoirs exceeding 0.49 Euro/kg. These figures are related to estimated transportation costs from farms to stationary abattoirs. According to Helgesson et al. [49], costs were estimated at 0.49 Euro/kg in northern Sweden and 0.31 Euro/kg in southern Sweden. Although the survey generally states that people might be willing to pay extra for enhanced animal welfare, it does not state if people are willing to accept a higher—i.e., a negative—impact on the environment, which is a possible outcome as per our results. This observation should be further analyzed. Miranda-de la Lama et al. [50] report a high interest in animal welfare issues among Mexican meat consumers; the authors state that they are willing to pay more for certified welfare-friendly products. The same interest is manifested in other international communities as demonstrated by Vanhonacker and Verbeke [51]. None of these interests, though, illustrate a conflict between animal welfare and environmental impact.

One of our analysis restrictions included a lack of information about the real-time routes and the type of truck used. To simulate these routes, we had to use the optimization model that likely provided us with an optimistic picture of effective transportation planning. More concretely, the Base scenario is better than reality when compared with other scenarios; hence, the potential for increased animal welfare and reduced costs is larger than our results demonstrate. In other words, the difference between optimized route planning and today’s manual planning should be larger than the results we illustrate. Future studies should include real-life data analyses and detailed routes to define the real potential of computerized optimization in animal transportation route planning.

The distance between farms and abattoirs is calculated with a route-finding optimization model developed for forestry transportation. The model finds the most viable route for timber transportation through information about road characteristics. The downside, however, is that the most viable route for animal transportation is most likely different from the route used to transport timber. Timber transportation routes do not have to consider route itineraries when carrying cargo from point A to point B. According to Algers et al. [46], some animal welfare factors are crucial when choosing routes between point A and point B. Some of these the number of traffic lights, roundabouts and curves. Aradom [52] and Gebresenbet et al. [53] point out that road quality and paving have an impact on animals’ journey experiences; these factors should preferably be included in further route planning development and analyses. Svenson et al. [54] describe, for example, the use of vertical and horizontal curvatures in route selection and distance calculation for logging trucks. Also, the risk of livestock truck accidents should be considered in route planning. Miranda-de la Lama et al. [55] analyzed the characteristics of livestock vehicle accidents in Spain over a nine-year period. Driver fatigue appeared to be one of the main causes of accidents as a result of poor route planning. Routes should be designed with animal welfare, economics, environment and driver’s capabilities in mind.

Optimizations authorized only one type of truck capacity. For this experiment, we had a capacity of fifteen cattle. We had one capacity in part because we obtained no information on the actual capacity of used trucks. In fact, cattle trucks across Sweden can transport up to fifty-five animals when equipped with a trailer and two floors. Many European countries and international communities are equipped with trucks having similar capacities when short-distance livestock transportation is common. [31, 34, 35, 56]. Our approach and optimization model should prove useful in a broader context: a future model development might include the possibility of varying truck sizes. Through this model it would be possible to analyze the effect on animal welfare, costs and emissions when using trucks larger than modern-day standards. According to Adell et al. [57], using larger trucks would reduce both transportation costs and energy consumption. This report is too general, though, and does not cover livestock transportation; it will require further investigation. For this analysis, we present relative figures when comparing different planning time windows and two transportation constraints; therefore, the truck size will not be heavily impacted.

Reducing slaughterhouses and live auction markets across Europe increase the journey times for animals [39, 58]. What’s more, cattle are not always transported to the closest abattoir, since competitions intensifies among slaughter companies when owners buy from farmers. According to Håkansson et al. [39], strategic planning in cattle transportation could reduce distance by 40% if cattle were always transported to the nearest abattoir. This potential is not currently utilized; slaughter companies attempt to create efficient routes with as little driving between farms as possible to reduce costs and environmental impact, all the while maintaining good animal welfare without regard to geographic possibilities. Future work should investigate route planning with computerized optimization, as we would see how planning is affected by possibilities of carrying out cattle exchanges among slaughter companies prior to transportation. This is common practice in Sweden’s forest industry [59]. Forest companies constantly compete with each other when buying wood from private forest owners, but they collaborate with each other before arranging transportation. When all parties have reached a deal with the forest owner, they make ensure that wood is allocated to the closest mill.

## Conclusions

Unlike today’s manual planning, we have developed and tested a computerized route optimization model for animal transportation capable of pointing out measures that substantially improve animal welfare with reduced transportation times and number of stops. We have shown that changes in regulations, such as minimizing the number of allowed stops along each route or reducing animal transportation time, will have positive effects on animal welfare. Further, this will result in increased working times and driving distances, leading to higher transportation costs and a negative environmental impact. Deciding which alternative is the most desirable in each case is difficult, but can be supported by the route optimization model that offers the possibility of testing different alternatives beforehand.

Future model developments for improving animal welfare include minimizing queuing time and today’s extensive need for keeping animals at the abattoirs over-night. These would require developing more detailed routing models. Improvements can be reflected in including road quality details such as vertical and horizontal curvature, braking, acceleration and deceleration needs.
